# Prescriptions and Dispensing

**Published:** 1908-11-28

**Authors:** 


### Therapeutics.

### PRESCRIPTIONS AND DISPENSING.

### COMPRESSED TABLETS (*continued*).

Potato starch, known commercially as farina, and arrowroot starch are more efficient than other kinds, and one of these two should be employed. The amount that is necessary varies in different cases, between 5 and 10 per cent. If any addition of the kind is made at all, it is best to add enough to ensure the object in view (disintegration of the tablet), even if as much as 1 grain of starch has to be added to each tablet.

Phenacetin and similar materials must be finely powdered first, and the starch then added. The powder must then be moistened, and various liquids are suitable for this purpose in different cases, the principal being water, spirit, and very weak solutions of sugar, gum, or dextrin. Many substances can be granulated quite well with plain water or spirit, and when an adhesive substance like dextrin is required the smallest possible quantity should be used. All that is required is to prevent the granules falling to powder after drying, when transferred from vessel to vessel or subjected to the vibration of the hopper of the machine. The powder having been damped with no more of the liquid than is just sufficient to give to it a clinging character, it is gently passed through a No. 20 sieve, when it will come through in the form of small moist granules; it is then spread out in a thin layer to dry in a fairly warm place. When dry it may with advantage be again passed through a No. 20 sieve, to break down any portions that may have become aggregated into larger masses.

A lubricant must now be added, in order that it may run easily. Finely powdered French chalk (talc) is most often employed, and from 1 to 3 per cent. is usually required. This is scattered over the granules in a thin layer, and the whole then gently shaken in a dry bottle or other vessel. The material is then ready for compression.

Other lubricants besides French chalk may be used. If the material of the tablets is soluble in water, it is not desirable to add an insoluble substance; boric acid can then be employed. Pure liquid paraffin can also be used, this being sprayed on to the granules with a fine spray. A solution of white soft paraffin in ether is preferred in other cases, the granules then being exposed to the air after spraying until all the ether has evaporated.

Oil of theobroma is also very useful, and a convenient method has been devised by which granulation and lubrication with this oil can be done in one operation. An emulsion of oil of theobroma is prepared by the aid of soap and a little gum tragacanth, or gum acacia and gum tragacanth for cases in which soap would be objectionable, and the powder for compression is moistened with this emulsion and passed through a No. 20 sieve. Granules are formed by so doing, and these are dried by exposure to the air, after which they are ready for compression without further lubrication. When the material to be compressed is of such a nature that it would become unduly sticky when moistened with a watery liquid, a solution of oil of theobroma in ether and alcohol may be used in place of the emulsion; this has the further advantage that the granules dry more rapidly, and it is on that account more suitable in dispensing. The emulsion is, of course, preferable from the point of view of lower cost.

Many formulæ can be prescribed equally well as tablet or as pill, but it must not be supposed that, for making a tablet, a pill-mass can be made and then dried and compressed. A considerable degree of cohesion is required in a pill-mass, to permit of rolling, cutting, and rounding; and this has to be attained, as we have seen, by the use of a fair amount of moisture, or some sticky substance, such as an extract.

The pressure exerted in making a compressed tablet is so great that an apparently quite dry powder, if it contains an extract, is at once made perfectly coherent. The material that is prepared for compression requires the least possible amount of moisture to be added. Extracts should be dried and powdered, either alone or with some drying powder; all the ingredients should be finely powdered and mixed, then just moistened with a suitable liquid, and granulated. The granules should then be dried, lubricated, and compressed as already described.

The following is a comparative example of a prescription which might be made either into pills or into tablets:—

| | | |
|---|---|---|
| ℞ Aloin ... ... ... ... ... | | gr. xx. |
| Strychninæ ... ... ... ... | | gr. iss. |
| Extracti Belladonnæ ... ... | | gr. xij. |
| Excip. q.s. ... ... ... | ad | gr. c. |

Misce. Fiant pilulæ vel tabellæ 100.

If pills are to be made, the 66½ grains of excipient would be about 30 grains of powdered liquorice, and the remainder confection of roses or some other binding material. If, on the other hand, tablets are to be made, the whole 66½ grains should be milk sugar. After thorough mixing, enough water is added to make the powder just moist; it is then passed through a sieve, forming granules, which are dried and lubricated, and are then ready for the hand-machine.

Tablet-making, like all dispensing, requires practice before proficiency in it can be attained; before leaving the subject, however, two general rules may be mentioned, due observance of which will go far to enable even a beginner to turn out fair tablets. These are:—

1. Do not compress the granules that are not properly dried; slightly moist granules do not feed well from the hopper, and they are very prone to stick to the punches.

2. Remember that it is impossible to turn out tablets with a good finish if the surfaces of the punches are at all rough; it is not only necessary to clean and dry them thoroughly after each using, but they should be well polished with the finest emery at short intervals.

In our next article we will pass on to the question of confections, pastilles, and pastes.

